# A Measurement Science Framework to Optimize CDS for Opioid Use Disorder Treatment in the ED

**DOI:** 10.1055/a-2595-0317

**Published:** 2025-09-12

**Authors:** Mark S. Iscoe, Carolina Diniz Hooper, Deborah R. Levy, John Lutz, Hyung Paek, Christian Rose, Thomas Kannampallil, Daniella Meeker, James D. Dziura, Edward R. Melnick

**Affiliations:** 1 Department of Emergency Medicine, Yale University School of Medicine, New Haven, Connecticut, United States; 2 Department of Biomedical Informatics and Data Sciences, Yale University School of Medicine, New Haven, Connecticut, United States; 3 Department of Veterans Affairs, Amarillo VA Healthcare System, Amarillo, Texas, United States; 4 Yale University School of Medicine, New Haven, Connecticut, United States; 5 Department of Biostatistics (Health Informatics), Yale School of Public Health, New Haven, Connecticut, United States; 6 Department of Emergency Medicine, Stanford University School of Medicine, Stanford, California, United States; 7 Institute for Informatics, Data Science, and Biostatistics Washington University School of Medicine, St. Louis, Missouri, United States

**Keywords:** emergency medicine, addiction, workflow, measurement and observation, electronic health records and systems

## Abstract

**Objectives:**

In the emergency department-initiated buprenorphine for opioid use disorder (EMBED) trial, a clinical decision support (CDS) tool had no effect on rates of buprenorphine initiation in emergency department (ED) patients with opioid use disorder. The Agency for Healthcare Research and Quality (AHRQ) recently released a CDS Performance Measure Inventory to guide data-driven CDS development and evaluation. Through partner co-design, we tailored AHRQ inventory measures to evaluate EMBED CDS performance and drive improvements.

**Methods:**

Relevant AHRQ inventory measures were selected and adapted using a partner co-design approach grounded in consensus methodology, with three iterative, multidisciplinary partner working group sessions involving stakeholders from various roles and institutions; meetings were followed by postmeeting surveys. The co-design process was divided into conceptualization, specification, and evaluation phases building on the Centers for Medicare and Medicaid Services' measure life cycle framework. Final measures were evaluated in three EDs in a single health system from January 1, 2023, to December 31, 2024.

**Results:**

The partner working group included 25 members. During conceptualization, 13 initial candidate metrics were narrowed to 6 priority categories. These were further specified and validated as the following measures, presented with preliminary values based on the use of the current (i.e., preoptimization) EMBED CDS: eligible encounters with CDS engagement, 5.0% (95% confidence interval: 4.3–5.8%); teamwork on ED initiation of buprenorphine, 39.9% (32.5–47.3%); proportion of eligible users who used EMBED, 58.3% (50.9–65.8%); time spent on EMBED, 29.0 seconds (20.4–37.7 seconds); proportion of buprenorphine orders placed through EMBED, 6.5% (3.4–9.6%); and task completion, 13.8% (8.9–18.7%) for buprenorphine order/prescription.

**Conclusion:**

A measurement science framework informed by partner co-design was a feasible approach to develop measures to guide CDS improvement. Subsequent research could adapt this approach to evaluate other CDS applications.

## Background and Significance

Motivated by the opioid crisis and the opportunity to increase initiation of buprenorphine, a life-saving 1 2 but underutilized 3 medication for treatment opioid use disorder (OUD) in the emergency department (ED), 2 4 5 6 we developed and implemented the emergency department initiated buprenorphine for opioid use disorder (EMBED) clinical decision support (CDS) tool. 7 8 While CDS has the potential to augment clinical care, the effectiveness of an individual intervention can be limited or incremental. 9 Although more eligible physicians initiated buprenorphine after EMBED implementation, patient-level initiation did not increase. 10 We therefore sought to improve our CDS tool.

Data-driven improvement of electronic health record (EHR) tools including CDS requires scientifically sound measurement to inform targeted improvements. 11 Yet standardized metrics for EHR use are not widely adopted, 11 12 13 impeding our understanding of how CDS influences care. 14 15 Moreover, generalized measures do not account for organizational, clinical, and individual user contexts. 11 14 A new novel tool for addressing this deficit is the Agency for Healthcare Research and Quality's (AHRQ) Patient-Centered CDS Performance Measurement Inventory (hereafter referred to as the AHRQ Inventory), a library of context-specific CDS use measures developed via a scoping review process. 16 This list of 163 measure constructs, categorized by implementation phase and domain addressed, is intended to be adapted to fit a specific CDS's evaluation needs. Partner or collaborative co-design, the method of engaging partners (traditionally referred to as “stakeholders”) throughout the process of developing and refining an intervention, 17 can help ensure that the measures are adapted in a way that is contextually informed and meets user, patient, and other stakeholder needs 18 19

## Objectives

To improve the use and effectiveness of the EMBED CDS, we adapted items from the AHRQ inventory using a multidisciplinary, iterative, partner co-design approach. In the upcoming Adaptive Decision Support for Addiction Treatment (ADAPT) multiphase optimization strategy (MOST) 20 trial (ClinicalTrials.gov Protocol #NCT06793696), these novel measures will be implemented in a series of rapid, randomized tests to assess CDS efficacy and ultimately improve EMBED, increasing buprenorphine initiation in appropriate ED patients with OUD. The full protocol for the ADAPT trial has been described previously. 21

## Methods

**Fig. 1:**
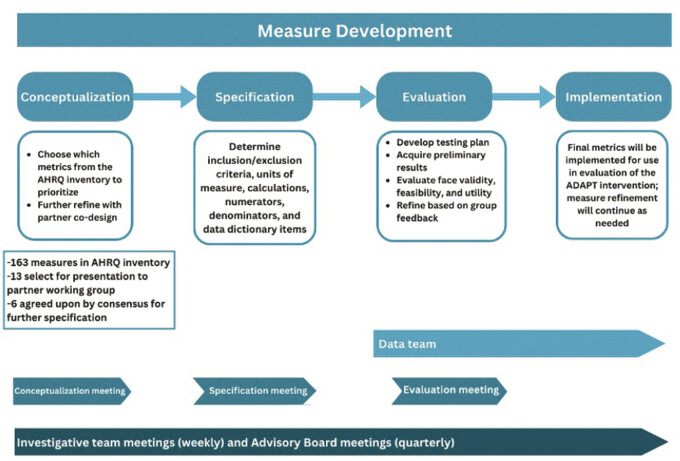
Visual overview of the measure development process.

We employed existing frameworks to develop scientific measures of CDS use. Adapting the Centers for Medicare and Medicaid Services (CMS) measure lifecycle framework (conceptualization, specification, implementation, testing, use, evaluation, and maintenance), 22 we divided our measure development process into phases of conceptualization, specification, and evaluation; while the phases were conducted successively, we employed a lifecycle approach where feedback or insights from later phases informed reevaluation or refinement of conclusions from earlier stages ( Fig. 1 ). We applied a modified Delphi 23 approach in each phase of measure development, through a collaborative co-design framework. The modified Delphi technique can be used to incorporate the perspectives of key partners and subject matter experts in health technology initiatives. 19 24 25 Finally, we adhered to best practices in using EHR audit log data for research: (1) establishing transparent and standardized measures; (2) thinking broadly about EHR work and including measures of teamwork, and (3) linking measures to patient-level clinical endpoints. 12

The measures were preliminarily applied at 3 EDs in a single health system: an academic level 1 trauma center; an urban community ED; and a suburban, freestanding ED. All sites use the same instance of the Epic EHR (Epic Systems; Verona, Wisconsin, United States ).

### Consensus Approach

Partner working group members were recruited via email with the goal of establishing a diverse group of stakeholder partners with different backgrounds across roles and settings locally and nationally. With a goal of creating measures that collectively capture key user- and patient-centered aspects of CDS with “importance, scientific soundness, usability, and feasibility,” 26 partners were convened in three virtual meetings. Each meeting was conducted over Zoom (Zoom Communications; San Jose, California, United States), lasted approximately 120 minutes, and included three segments: (1) introductions with background, level setting, and refreshers, (2) break-out working sessions in three sub-groups with a specific set of tasks relevant to the development phase, and (3) debrief with preliminary consensus using group discussions and real-time surveys addressing alternative specifications, prioritization, and implementation options. Following each meeting, postmeeting surveys addressing outstanding questions were distributed via email to all partner working group members and completed through Qualtrics (Qualtrics; Provo, Utah, United States).

### Measure Development

Prior to measure conceptualization, each measure in the AHRQ Inventory was reviewed by the investigative team and graded on its potential applicability to the EMBED CDS.

In the conceptualization meeting, we introduced group members; provided an overview of the opioid epidemic and the role of buprenorphine in treating patients with OUD; summarized ED workflows for nonclinician group members; described the functionality of the EMBED CDS along with its prior successes and failures; summarized the upcoming ADAPT trial; defined the stages of the objective of measure development; and presented our 13 preliminary measure categories. The working group then reviewed each candidate measure in-depth, identifying potential strengths and limitations as well as considerations for meaningful application; after discussion, the group came to a preliminary consensus on which measures to prioritize, through Likert scale ratings. In the postmeeting survey, partners individually ranked each candidate measure on the same scale based on a summary of the groups' comments and the group's preliminary vote.

For the specification meeting, we described potential EHR data elements and sources; discussed the purpose of each candidate measure as a group, including potential shortcomings of EMBED that the measure could track; and discussed each measure's potential relevance to user- and patient-centered outcomes. Our approach to the discussion was guided by McGlynn's assertion that measures should be assessed on the criteria of “importance, scientific soundness, usability, and feasibility.” 24 With this background and motivation, we split again into three breakout groups, which each worked on the specification of two measures, or the task of creating clear directions for how each measure should be calculated. Specification entailed defining precise inclusion and exclusion criteria, data source(s), and specific values to be calculated including numerators and denominators for ratio variables. With a goal of harmonization of these definitions, in our postmeeting survey, we sought direct feedback on any outstanding specification questions or areas in which group members expressed divergent opinions.

Finally, in our evaluation meeting, we presented our preliminary calculations of measures based on current specifications; presented obstacles or ambiguities identified in the calculation process; solicited feedback on specific outstanding questions; sought domain experts' opinions on measures' face validity; and discussed each measure as a group, with a focus toward future refinements. In contrast to other meetings, we did not use breakout groups in this meeting, because we wanted each partner to have the opportunity to provide their perspective on each measure. The final postmeeting survey presented working group members with outstanding questions where full consensus (defined as 80% or greater agreement across group members) had not yet been reached.

### Initial CDS Measure Values

All measures were constructed from data available in our institution's local EHR database. Potential data elements, which were presented to working group members in the measure conceptualization and specification phases, included patient care team composition, EHR users' responses to alerts, users' action- and time-based interactions with the CDS extracted from audit log data (Epic's User Action Log Lite and Access Log), flowsheet completion, orders entered, patient diagnoses and discharge materials, and whether a patient had been flagged as potentially having OUD, based on a computation phenotype described previously. 27 Baseline measure values describing the use of the existing EMBED CDS between January 1, 2023, and December 31, 2024, were reported using descriptive statistics (means and proportions) with 95% confidence intervals.

## Results

The partner working group consisted of 25 members. Members represented eight different institutions and included (with some members having expertise in multiple domains): emergency medicine physicians (8), addiction medicine physicians (2), a clinical toxicologist, ED clinical pharmacists (2), nurses (3), an ED health promotion advocate (addiction counselor), clinical informaticists (10, including 6 with expertise in EHR use measurement), EHR analysts and programmers (4), data scientists (2), a national EHR vendor representative, and an emergency medicine specialty society's quality initiative representative.

From 163 initial AHRQ Inventory measures, the investigative team identified 13 that were likely to provide valuable and distinct insights into the EMBED CDS's use and usability. In the conceptualization phase, the partner working group prioritized 6 of these preliminary measures for further specification ( Table 1 ).

**Table 1:** Preliminary measures adapted from the AHRQ inventory for partner working group consideration in the conceptualization phase with summarized group feedback

AHRQ measures construct category	AHRQ measures construct description	Measure as presented in conceptualization partner working group meeting	Summary of partner working group feedback	Corresponding final measure (if selected; details in Table 2 )
Alert fatigue	Alerts received per encounter and comorbidity index of the clinician's patients	Cognitive load/patient complexity	–Complex to calculate –May reflect more about ED workflow than CDS itself	
Alert fatigue	Time interval between the appearance of the alert and the completion of the selected actions	Time elapsed from alert to prescription	–There are many confounding factors at play that may not be captured by this metric –Does not allow us to distinguish between EHR workflow and ED clinical workflow	
CDS implementation integrity	Number of alerts overridden (alerts suppressed)	Ratio of alerts dismissed	–Timing of alert dismissal could help drive CDS logic –Should be considered in the context of alert trigger criteria –Could provide more useful information if paired with prescribing data	Proportion of eligible encounters with CDS engagement
CDS implementation integrity	Ratio of alerts to order	Ratio of alerts to orders/prescriptions	–Requires careful consideration of who is included in the numerator and denominator; for example, how to consider cases in which the alert does not fire but the clinician seeks out the CDS –More meaningful if paired with time spent on CDS	
Clinician workflow integration	Frequency of task switching and interruptions (workflow fragmentation)	Frequency of task switching and interruptions	–Cannot capture non-EHR interruptions –Difficult to interpret without clinical context of interruption(s)	
Clinician workflow integration	Level of teamwork within the care team	Teamwork on CDS	–May help guide CDS refinements that empower various team members –Will need to define potential roles (e.g., nurse to complete COWS score)	Teamwork on ED initiation of buprenorphine
Clinician workflow integration	Task completion rate	Task completion rate	–Important to know when CDS is started but not completed –Challenging to calculate; might need a decision tree to understand whether the appropriate pathway was completed –Would be helpful to know which specific tasks are associated with buprenorphine orders/prescriptions, allowing prioritization of high-value items	
Clinician workflow integration	Task completion rate	Task abandonment	–Could help improve efficiency –Could help identify bottlenecks in multi-step CDS workflow –It would be important to know if a prescription was sent despite CDS being abandoned (i.e., is the full CDS needed)	Task completion (with sub-measures for each element)
Clinician workflow integration	Time spent by a clinician on a continuous task (“time on task”)	Time spent on CDS (or CDS elements)	–Given ED time constraints, the time spent on CDS may affect the likelihood of using the CDS again –On the other hand, zero or minimal time may indicate an issue with the CDS	Time spent on EMBED (or EMBED elements)
Policy and safety compliance	Ratio of orders submitted using a specific research protocol or set of standard care prescriptions	Ratio of orders placed through CDS vs. externally	–Simple and valuable, this is the only metric that shows when clinicians are treating OUD but not using the CDS –Could help promote care safety	Proportion of encounters in which buprenorphine was ordered through EMBED vs. externally (i.e., through other EHR workflows)
Policy and safety compliance	Use error rate	Order error rate (e.g., retract-and-reorder)	–Rare event and may in fact never occur for orders placed through the CDS	
Reach	Proportion of eligible providers who interacted with the CDS system at least once	Proportion of users who used the CDS	–Straightforward metric that provides information not captured elsewhere	Proportion of eligible users who used EMBED
Software performance	Binary outcome across data categories where users either successfully retrieved all information or failed to retrieve all information	Retrieval and display of data	–Very challenging to calculate accurately –Because we don't know if the information that is displayed is needed, it's hard to know the significance of this metric –Limited patient-level relevance	

**Fig. 2:**
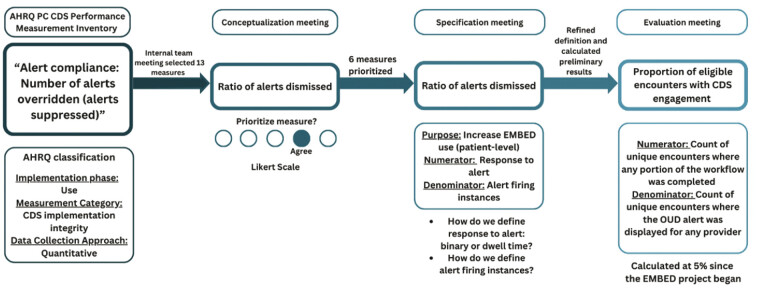
Example of iterative development of one measure. Thirteen measures from the AHRQ inventory were selected by the project leadership based on pertinence to the EMBED intervention. After reviewing each preliminary measure, the working group reached a consensus on which measures to prioritize in the conceptualization meeting. In the specification meeting, inclusion and exclusion criteria, data sources, and formulas were defined for each selected measure. Subsequently, preliminary results were calculated and reviewed at the evaluation meeting. Finally, ambiguities and outstanding questions were clarified.

In the specification and evaluation phases, each of the six prioritized measures was preliminarily specified, further refined with discrete formulae including numerators, denominators, and reporting stratification as appropriate, and preliminarily calculated ( Table 2 ). Fig. 2 visualizes the refinement of one measure through each development phase.

**Table 2:** Final measures along with intended purpose, the abbreviated formula for calculation, and preliminary value based on the use of the current EMBED CDS from January 1, 2023, to December 31, 2024

Measure	EMBED shortcoming addressed	Purpose	Formula (brief)	Baseline value (95% CI)
Proportion of eligible encounters with CDS engagement	Infrequent CDS use (launched in only 9.4% of eligible encounters in the initial EMBED trial) a	Increase CDS engagement	Numerator: Encounters in which any portion of the EMBED workflow (OUD diagnosis, withdrawal assessment, readiness assessment, buprenorphine or adjunct medication order/prescription, postvisit referral order) was documented or completed Denominator: Encounters in which an alert fired	5.0% (4.3–5.8%)
Teamwork on ED initiation of buprenorphine	No direct involvement of nursing in CDS workflow b	Increase collaborative, team-based care	Numerator: Encounters in which more than one team member (regardless of role) helped complete the EMBED workflow (by definition above) Denominator: Encounters in which at least one user engaged with the EMBED CDS (see comment above)	39.9% (32.5–47.3%)
Proportion of eligible users who used EMBED	Poor reach (launched by only 38.2% of physicians in the initial EMBED trial) a	Increase EMBED use on a user level	Numerator: Staff members across roles who used EMBED (by definition above) at least once during the study period Denominator: Staff members who cared for at least one patient for whom the alert fired during the study period	58.3% (50.9–65.8%)
Time spent on EMBED (or EMBED elements)	UI or workflow challenges may deter the completion of the EMBED workflow	Understand EMBED use patterns and identify inefficiencies	Time (in seconds) spent on EMBED overall and by component	29.0 s (20.4–37.7 s) c
Proportion of encounters in which buprenorphine was ordered through EMBED vs. externally	Users who are (a) unfamiliar with or uninterested in EMBED or (b) experience with buprenorphine prescription and dosing may avoid CDS, risking inappropriate dosing	Promote the use of EMBED order sets in order to provide safe, effective, guideline-concordant care	Numerator: Encounters in which buprenorphine order is placed through EMBED (i.e., using EMBED order set) Denominator: Encounters in which buprenorphine is ordered and is NOT on the home med list	6.5% (3.4–9.6%) d
Task completion (with sub-measures for each element)	EMBED workflow was often abandoned before buprenorphine prescription	Identify work patterns and obstacles to workflow completion	Numerator(s) with criterion for task completion, on the encounter level: •COWS: Score entered •OUD diagnosis: Diagnosis recorded •Readiness assessment: readiness indicated •Buprenorphine order/prescription: Any buprenorphine order or prescription placed Denominator: (shared across all sub-measures): EMBED opened	COWS score: 15.9% (10.7–21.1%) OUD diagnosis: 7.4% (3.7–11.1%) Readiness assessment: 1.1% (0.0–2.5%) Buprenorphine order/prescription: 13.8% (8.9–18.7%)

a Indicating patient had been identified as potentially having OUD.

b Nurses do not receive alerts in the current iteration of the EMBED CDS.

c Value refers to the total time spent on EMBED; the measure can be further stratified by clinical role and by what is accomplished in the encounter.

d Value refers to the proportion of eligible encounters in which EMBED was used for at least one buprenorphine order; the proportion of encounters in which EMBED was used for all buprenorphine orders is 4.4%.

## Discussion

In this case study, we demonstrate how partner co-design methods can be used to adapt AHRQ Inventory 16 measures to a specific underperforming CDS instance around the treatment of OUD in the ED. In the literature on CDS refinement and evaluation, stakeholder involvement and quantitative CDS assessment have typically been distinct domains. Prior initiatives, for example, have used qualitative stakeholder input and collaboration to inform the iterative development of CDS tools 7 10 28 29 30 31 32 33 34 35 36 37 38 And while others have relied on clinical and operational endpoints to assess CDS efficacy, 39 40 these evaluations rarely incorporate detailed analyses of CDS usage patterns. 41 42 For example, studies evaluating CDS for safe opioid prescribing 7 36 37 42 43 or treatment of OUD 10 have typically focused on prescription rates as primary outcomes. Our approach differs by integrating stakeholder collaboration to guide the selection and development of CDS evaluation measures using the new AHRQ Inventory. This methodology allows us to move beyond outcomes of prescription rates and binary CDS usage measures (i.e., whether the CDS was used or not) to incorporate measures such as time spent interacting with CDS and teamwork dynamics, providing a more comprehensive assessment of CDS functionality and effectiveness. This replicable model could be applied in similar cases where CDS refinement is needed. Scientifically sound and collaboratively designed measures such as these could be implemented to track CDS use and effectiveness, identify weaknesses, and, as they will in the ADAPT trial, compare different CDS iterations to drive refinements.

### Lessons Learned

We found that having an interdisciplinary partner group with diverse backgrounds, experiences, and perspectives provided valuable insight into measure development, shaping measures corresponding to their areas of expertise. In particular, members of the patient care team (physicians, nurses, addiction counselors, and clinical pharmacists) were able to provide context relevant to McGlynn's criteria 26 of scientific soundness (from a clinical perspective) and usability; informaticists, programmers, and data scientists provided complementary insight on scientific soundness (from a technical perspective) and feasibility. For example, informaticists noted limitations in a proposed measure related to task-switching and interruptions, since measurement of clinical interruptions that occurred outside of the EHR (e.g., an overhead page related to a critical patient) would be infeasible in a project of this scale. Conversations and questions among group members helped to elucidate importance, ensuring that measures captured relevant information with the potential for meaningful variance; for example, the health promotion advocate shared that time spent on CDS, which was initially conceptualized as a measure of clinician burden, was also important to patients with OUD, for whom a delay in opioid withdrawal treatment could be agonizing. These conversations helped align the group's focus and define the goals of the project, finding a middle ground or overlap between the ideal and the feasible.

Further, we found that CMS's measure life cycle framework 22 complemented the partner co-design approach. Partners provided valuable contributions in each step of the iterative measure development process, from conceptualization to evaluation. Had they only been included in one phase of the project, it would have been challenging to adequately incorporate their input. As shown in Figs. 1 and 2 , measures evolved throughout the development process. Even in cases where conceptualization and specification initially seemed straightforward, we found that partner review of preliminary measure calculations in the evaluation meeting was informative. For example, upon reviewing preliminary calculations, the working group noted that the measure capturing whether burpenorphine was ordered through EMBED or externally, which had initially been calculated per order (i.e., the proportion of buprenorphine orders placed through EMBED vs. externally) would be more informative if calculated on the encounter level (i.e., the proportion of encounters in which buprenorphine was ordered through EMBED). After further discussion, consensus was reached to modify this specification. Despite partners' involvement throughout the development cycle, participation only required six total meeting hours (two per session) and approximately 30 minutes of postmeeting surveys (10 minutes each) over the course of a year with the investigative team progressing independently in the months between meetings.

Finally, we should note that the measure development cycle helped to identify shortcomings of the current EMBED CDS and inspire potential improvements. For instance, in constructing our measure relating to teamwork on ED initiation of buprenorphine, we realized that, although nurses play a crucial role in the EHR-based care of patients with OUD, including COWS score documentation and calculation, they were not receiving alerts related to their patients' potential for withdrawal. In the upcoming ADAPT trial, we will therefore assess the effectiveness of a COWS reminder for nursing.

### Limitations

Our methodology has several limitations. First, while we have identified preliminary measures based on our existing CDS, we have not yet evaluated their impact on CDS optimization. This prospective assessment will occur over the coming years as part of the ADAPT trial. 21 While this is a key limitation, we believe that documenting the collaborative process of adapting the AHRQ inventory for a specific CDS tool provides valuable insights for others seeking to apply this framework in similar contexts. Second, it required significant time and resources (e.g., honoraria for nonstudy site working group members, schedule alignment for virtual synchronous meetings, and survey tool deployment for postmeeting surveys) that may not be available to all CDS optimization initiatives. We propose this methodology still has potential generalizability and scalability and could be integrated into existing CDS improvement efforts. 44 Finally, we demonstrated a single use case and our methods may not be equally applicable in optimizing other CDS interventions. Notably, the AHRQ inventory includes 163 measure constructs, some of which were not relevant to this CDS tool but may apply to others (e.g., CDS costs, clinician or patient knowledge, and software performance). Our selection prioritized constructs aligned with EMBED's automation and workflow integration goals, though the full inventory remains adaptable to diverse CDS implementations. We aim to continue to “fail forward” by not only using these measures to differentiate among various CDS iterations but also by evaluating the measures themselves and further refining them as necessary, with guidance from our working group.

## Conclusion

We found that a collaborative co-design approach using measurement science and iterative, multidisciplinary sessions allowed the adaptation of the AHRQ CDS Performance Measure Inventory to an underutilized CDS for the treatment of patients with OUD in the ED. Our 6 EHR-derived measures of CDS use collectively capture key user and process outcomes that can feasibly compare different CDS iterations in an upcoming clinical trial, with the goal of improving CDS usability, efficiency, and utility and facilitating safe, evidence-based buprenorphine initiation in appropriate ED patients. Ultimately, we aim to scale the optimized CDS package locally and nationally with the goal of adaptation to other EHR vendor products. This approach can be modified for other CDS domains and future work should explore its real-world effects on CDS use.

## Clinical Relevance Statement

This study outlines the iterative, interdisciplinary process through which we adapted the AHRQ CDS Performance Measure Inventory to develop meaningful measures to evaluate the EMBED CDS and improve its performance. This method could orient the systematic creation of metrics to evaluate CDSs in other contexts, but will ultimately aid our team in optimizing the EMBED CDS and increase buprenorphine prescription in the ED.

## Multiple-Choice Questions

1. Which factor commonly limits efforts to refine clinical decision support (CDS) tools through a scientifically rigorous approach?
  - EHR data are uniform across vendors.
  - Broad agreement exists on the best metrics to measure EHR use.
  - Standardized metrics for EHR use are not widely adopted.
  - Organizational, clinical, and user contexts are irrelevant to improvement.
  **Correct Answer:** The correct answer is option c. Standardized metrics for EHR use are not widely adopted. While data from EHR and CDS tools could guide targeted improvements, the lack of widely adopted, standardized metrics restricts the ability to create scientifically sound measures, as it becomes difficult to compare and interpret results across different settings. Establishing consistent standards that adapt to varied contexts is crucial for meaningful improvements in CDS.
2. What is one key advantage of a partner co-design approach in developing new CDS measures?
  - It requires no feedback from frontline clinicians.
  - It expedites the process by assigning decisions to a single expert.
  - It limits changes to the original design to avoid confusion.
  - It integrates diverse perspectives to ensure the measures are relevant.
  **Correct Answer:** The correct answer is option d. It integrates diverse perspectives to ensure the measures are relevant. A partner co-design approach includes stakeholders with various roles collaborating throughout the process. This diversity promotes the creation of CDS measures that align closely with real-world needs, enhancing their relevance, validity, usability, and feasibility.
